# Multicenter development and validation of a serum microRNA–based diagnostic model for breast cancer and its association with tumor burden and biological aggressiveness: an observational case-control study

**DOI:** 10.1186/s13058-026-02361-1

**Published:** 2026-08-06

**Authors:** Ayane Yamaguchi, Kana Kawanishi, Kei Ishii, Yukiko Fukui, Hanako Shimizu, Masahiro Kawashima, Ryo Otomo, Soseki Taniguchi, Mitsuharu Hirai, Wakako Tsuji, Sachiko Takahara, Hiroyasu Yamashiro, Hirotsugu Yanai, Yoshio Moriguchi, Shigeru Tsuyuki, Mariko Nishie, Hirofumi Suwa, Norikazu Masuda, Masakazu Toi

**Affiliations:** 1 Department of Breast Surgery, Kyoto University Graduate School of Medicine, Kyoto, Japan; 2 Research and Development Division, ARKRAY, Inc., Kyoto, Japan; 3 Department of Breast Surgery, Shiga General Hospital, Shiga, Japan; 4 Department of Breast Surgery, Kitano Hospital, Osaka, Japan; 5 Department of Breast Surgery, Tenri Hospital, Nara, Japan; 6 Department of Breast Surgery, Kansai Medical University Hospital, Osaka, Japan; 7 Department of Breast Surgery, Kyoto City Hospital, Kyoto, Japan; 8 Department of Breast Surgery, Osaka Red Cross Hospital, Osaka, Japan; 9 Department of Breast Surgery, Kyoto Katsura Hospital, Kyoto, Japan; 10 Department of Breast Surgery, Hyogo Prefectural Amagasaki General Medical Center, Hyogo, Japan; 11 Department of Breast Surgery, Tokyo Metropolitan Komagome Hospital, Tokyo, Japan

**Keywords:** Breast cancer, microRNA, Liquid biopsy, Biomarker, Machine learning, Diagnostic model, Multicenter study

## Abstract

**Background:**

Although mammography remains the cornerstone of breast cancer screening, its diagnostic performance is influenced by factors such as breast density and tumor characteristics. Blood-based biomarkers may therefore provide complementary biological information to imaging-based detection strategies. While circulating microRNAs (miRNAs) have emerged as promising minimally invasive biomarkers, their diagnostic performance and associations with clinicopathological characteristics remain incompletely understood. This multicenter study aimed to develop and validate a serum miRNA–based diagnostic model for breast cancer and to evaluate its associations with clinical stage, histological grade, and imaging-related factors.

**Methods:**

In this multicenter observational case-control study, serum samples from 345 patients with breast cancer and 373 cancer-free controls were analyzed using next-generation sequencing–based miRNA profiling. After normalization and batch correction, 114 stably expressed miRNAs were selected for model construction. An ensemble machine learning model integrating five algorithms (Lasso, Ridge, support vector machine, Nu-SVM, and histogram-based gradient boosting) was developed using a training cohort (*n* = 569) and evaluated in a held-out test cohort (*n* = 149). Model performance was assessed using receiver operating characteristic curve analysis and the area under the curve (AUC) with 95% confidence intervals calculated by the DeLong method. Model-derived prediction scores were further evaluated in relation to clinicopathological and imaging-related variables.

**Results:**

The serum miRNA–based diagnostic model demonstrated promising discrimination between breast cancer cases and cancer-free controls, with an AUC of 0.875 (95% CI, 0.820–0.930) in the held-out test set. Model-derived prediction scores increased with advancing clinical stage and showed an ordered trend across histological grades. In exploratory analyses, prediction scores tended to increase across higher clinical T categories and were higher among patients with distant metastasis, whereas no clear differences were observed according to mammographic category, breast density, or intrinsic tumor subtype.

**Conclusions:**

In this multicenter case-control study, a serum miRNA–based ensemble model demonstrated promising diagnostic discrimination for breast cancer. The model-derived prediction score was associated with clinical stage and histological grade, suggesting that circulating miRNA profiles may provide biologically relevant information beyond binary case-control discrimination. These findings support further investigation of serum miRNA profiling as a potential adjunctive diagnostic approach; however, prospective validation in representative screening populations is required before clinical implementation.

**Trial registration:**

Not applicable.

**Supplementary Information:**

The online version contains supplementary material available at https://doi.org/10.1186/s13058-026-02361-1.

## Background

Breast cancer is the most frequently diagnosed malignancy among women worldwide, and remains a leading cause of cancer-related morbidity and mortality [1, 2]. In recent years, although population-based screening programs have contributed to earlier detection and improved outcomes of breast cancer, participation rates in these programs remain suboptimal in many countries. In Japan, less than half of eligible women undergo regular breast cancer screening, a proportion lower than that observed in many other developed countries [3–5].

Mammography is the cornerstone of breast cancer screening and has been shown to reduce breast cancer mortality at the population level [6, 7]. However, its diagnostic performance is influenced by several factors, including breast density, patient age, and tumor characteristics. In particular, high mammographic breast density, which is common among women in their late 30s to 40s, reduces the sensitivity of mammography, and is itself an independent risk factor for breast cancer [8–11]. While adjunctive diagnostic modalities, such as ultrasonography, can improve cancer detection rates, especially in women with dense breasts, evidence regarding their impact on mortality reduction remains limited, underscoring the limitations of imaging-based strategies alone [11, 12].

Beyond imaging-related constraints, breast cancer is a biologically heterogeneous disease characterized by distinct tumor phenotypes and clinical behaviors. This heterogeneity is reflected in the existence of intrinsic tumor subtypes defined by hormone receptor and HER2 status, as well as in pathological features such as histological grade, which represents intrinsic tumor aggressiveness and is closely associated with proliferation, metastatic potential, and clinical prognosis [13–16]. Diagnostic approaches based solely on morphological assessment may therefore fail to fully capture the biological diversity of breast cancer.

Clinical stage reflects the extent of disease progression and tumor burden at the time of diagnosis, and remains one of the strongest determinants of outcomes and treatment strategy for breast cancer. From a diagnostic perspective, a critical unmet need is the ability to detect breast cancer at an early clinical stage, when tumor burden is limited and prognosis is most favorable. Blood biomarkers that can provide biologically informative and quantitative signals across disease stages, including early-stage disease, are therefore of particular clinical relevance.

Liquid biopsy has emerged as a promising approach to addressing these challenges, offering minimally invasive access to tumor-derived molecular information. Among circulating biomarkers, microRNAs (miRNAs) have attracted considerable interest due to their remarkable stability in blood and their regulatory roles in cancer-related biological processes. miRNAs are small, non-coding RNAs, typically 21–23 nucleotides in length, that modulate gene expression at the post-transcriptional level, and are involved in tumor initiation, progression, and interactions with the tumor microenvironment.

Accumulating evidence has demonstrated that specific circulating miRNA expression profiles differ between patients with breast cancer and healthy individuals, supporting their potential utility as diagnostic biomarkers [17–21]. However, the clinical translation of miRNA-based diagnostics has been limited by single-center study designs, modest sample sizes, and methodological heterogeneity across studies. Moreover, it remains unclear whether miRNA-based diagnostic indices behave as continuous biological markers that reflect disease burden, such as clinical stage, or whether they capture biological aggressiveness represented by pathological features including histological grade. In addition, although intrinsic tumor subtypes constitute important dimensions of breast cancer heterogeneity, their relationship with circulating miRNA profiles has been explored primarily in exploratory analyses.

In this multicenter study, we analyzed serum miRNA profiles from patients with breast cancer and cancer-free controls collected across multiple institutions, using a standardized miRNA measurement and analysis platform. The objectives of this study were to establish a serum miRNA–based discriminant model for breast cancer detection, and to evaluate its relationship with clinically relevant indicators of tumor burden and biological aggressiveness, particularly clinical stage and histological grade. Exploratory analyses were additionally conducted to assess the behavior of the model across intrinsic tumor subtypes. By evaluating diagnostic performance in a multicenter case-control cohort and examining the associations between model-derived prediction scores and clinicopathological disease characteristics, this study aimed to investigate the potential of circulating miRNAs as biologically informative diagnostic aids complementary to existing imaging-based strategies.

## Methods

### Study design

This was a multicenter observational case-control study, including prospectively and retrospectively collected samples, designed to evaluate the diagnostic performance of a serum miRNA–based classifier of breast cancer and its association with clinical stage, imaging findings, and pathological characteristics.

Women who were histologically diagnosed with breast cancer or scheduled to undergo histological diagnosis at Kyoto University Hospital and collaborating institutions were enrolled in this study. Patients were classified according to clinical stage at diagnosis into Stage 0, Stage I, and Stage II–IV groups, and were assigned to either a training set or a test set. Eligible patients were women aged 18 years or older. Patients were excluded if they had a history of malignancy, prior treatment for breast cancer, history of organ transplantation (including hematopoietic stem cell transplantation), severe psychiatric disorders that could interfere with informed consent, or any condition deemed inappropriate by the attending physician.

The non-cancer control group consisted of women with no self-reported history of cancer at the time of blood collection and no subsequent cancer diagnosis within one year. Mammography and ultrasonography were performed approximately one year after blood collection to exclude breast cancer. Serum samples and clinical information were previously collected by ARKRAY, Inc., a Japan-based medical device and diagnostic company headquartered in Kyoto, Japan. Eligible controls were Japanese women aged 20–89 years who provided written informed consent and for whom complete questionnaire data was available. Individuals with a current or past diagnosis of malignant disease, or those deemed unsuitable by the principal investigator were excluded. Control participants were confirmed to have no diagnosis of cancer one year after blood collection.

This study was conducted in accordance with the Declaration of Helsinki and was approved by the institutional review boards or ethics committees of all participating institutions. Written informed consent was obtained from all participants, or consent requirements were waived in accordance with institutional regulations.

### Blood sample collection and miRNA extraction from serum

Venous blood was drawn into serum-separating tubes. Following collection, serum was isolated by centrifugation and dispensed into cryotubes. The serum aliquots were frozen at − 80 °C until miRNA extraction. The interval between venipuncture and serum cryopreservation at − 80 °C was kept within the same calendar day, and any specimen exhibiting hemolysis was excluded from the study. Total RNA, including the miRNA fraction, was isolated from serum using the Maxwell^®^ RSC miRNA Plasma and Serum kit (Promega, AS1680). Purified miRNA samples were kept at − 80 °C until construction of the miRNA library.

Further details regarding pre-analytical sample processing consistency across participating institutions are provided in the Additional Methods.

### NGS library preparation and sequencing

Sequencing libraries were generated from the extracted miRNA using the QIAseq miRNA Library Kit (Qiagen, 331509) combined with the QIAseq miRNA 48 Index TF (96) (Qiagen, 331585) on an Agilent Bravo NGS liquid-handling platform (Agilent Technologies). Fragment size distribution and concentration of each library was assessed on a TapeStation HS D1000 system (Agilent Technologies, 5067–5584 and 5067–5585). After equimolar pooling, the libraries were loaded onto the Ion 540 Chip (Thermo Fisher Scientific, A27766) via the Ion Chef instrument (Thermo Fisher Scientific, 4484177), and sequencing was performed on the Ion GeneStudio S5 Prime or Plus platform (Thermo Fisher Scientific, A38196, A38195).

### miRNA annotation and quantification

Prior to miRNA annotation, the 5ʹ adapter sequences were trimmed from the raw sequencing reads using Cutadapt v4.6 [22]. The 5ʹ adapter-trimmed reads were processed using miRge 3.0 [23] for 3ʹ adapter trimming, quality filtering, alignment, and annotation. Reads were aligned to the human miRNA reference sequences in miRBase v22. The output read count matrix was used for downstream analyses.

### miRNA selection and normalization

Read count data from all detected miRNAs across all 718 samples were first subjected to reads-per-million (RPM) normalization to adjust for variation in sequencing depth among samples. The RPM values were then log2-transformed using the formula log2(RPM + 1), with a pseudocount of 1 included to handle zero counts. Next, 186 miRNAs that had a read count of ≥ 1 in at least 99% of all samples were selected from the raw expression matrix. Batch effects were corrected by applying ComBat from the R “sva” package [24] to the log2-transformed RPM data of these 186 miRNAs across all 718 samples jointly. Sequencing run was specified as the batch variable, diagnosis (breast cancer vs. non-cancer) was included as a biological covariate, and collection center was not included in the correction model. Consequently, batch-correction parameters were estimated using both the training and test samples.

For development of the breast cancer discrimination model, a more stringent filtration criterion was applied: 114 miRNAs that had a read count of ≥ 20 in at least 99% of all samples were selected from the raw expression matrix. This filtering step was based solely on predefined read-count and detection-frequency criteria and did not use case-control labels or diagnostic performance. The batch-corrected expression values of these 114 miRNAs (Additional file: Table 1) were used to construct the breast cancer discrimination model. Additional details regarding batch correction, quality control procedures, and principal component analysis for assessment of technical variation are provided in the Additional Methods.

### Construction of the breast cancer discrimination model

**Fig. 1:**
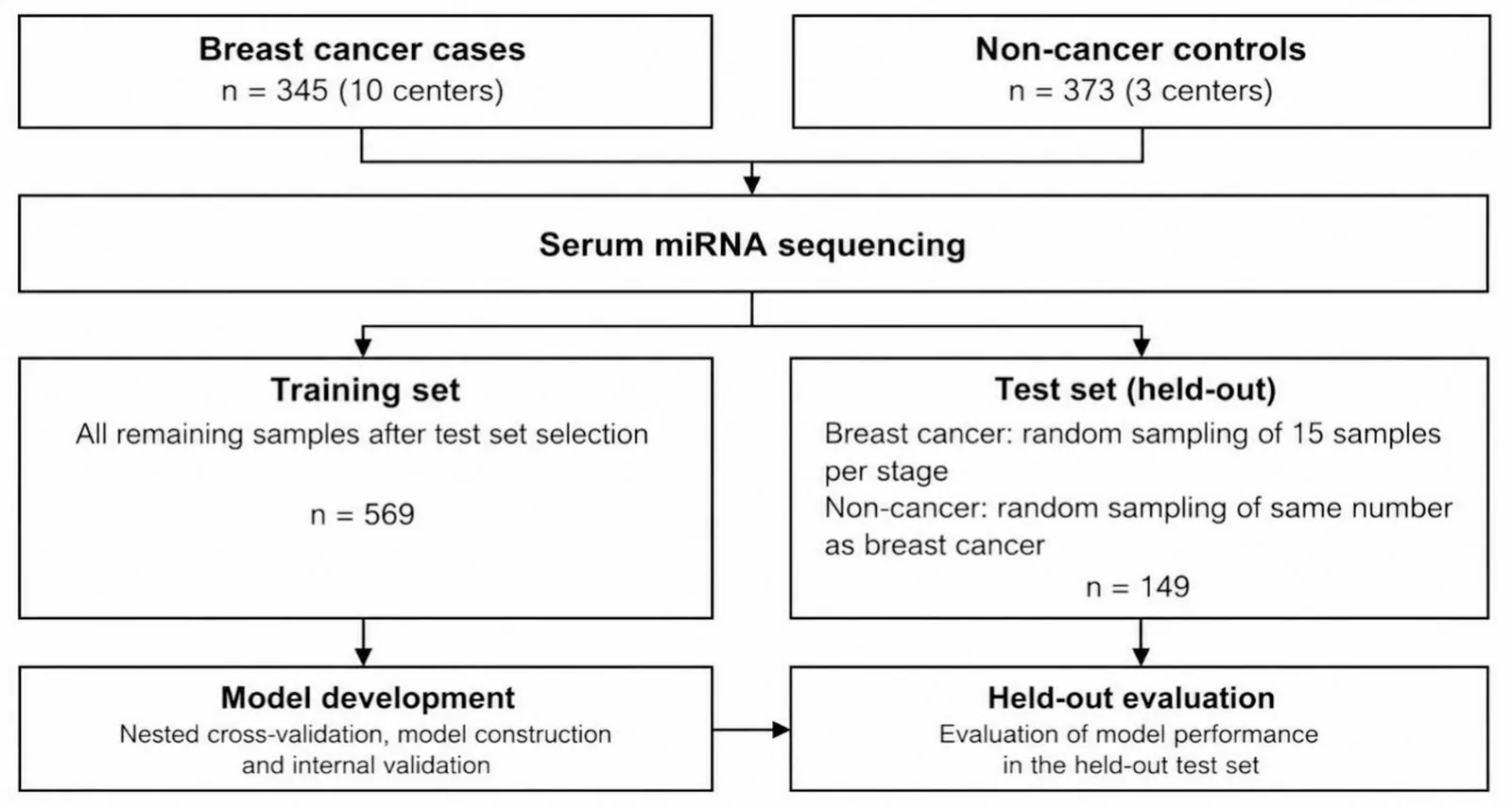
Flow diagram of the study cohort

To evaluate the generalization performance of the constructed model, participants were divided into a training set and a test set. From the 345 breast cancer patients, 15 patients were randomly selected from each of stages 0, I, II, and III, and these patients, along with all 14 stage IV patients (the total number available), were assigned to the test set, resulting in 74 breast cancer patients in the test set. Additionally, 75 non-cancer controls were randomly selected and assigned to the test set. The remaining 271 breast cancer patients and 298 non-cancer controls were assigned to the training set. Therefore, in total, the training set comprised 569 participants, and the test set comprised 149 participants (Fig. 1; Table 1).

Serum samples from patients with breast cancer and cancer-free controls were collected across multiple participating institutions. After applying predefined inclusion and exclusion criteria, eligible participants were assigned to the training set or the held-out test set used for model evaluation.

**Table 1:** Clinicopathological characteristics of breast cancer cases and non-cancer controls in the training and test sets

Variables		Training BC	Training control	Test BC	Test control
Sample size (n)		271	298	74	75
Age in years, median (IQR)		59 (49–73)	57 (44–71)	55 (50–66)	57 (44–71)
Menopausal status	Premenopausal	82	79	23	21
	Postmenopausal	184	85	49	19
	NA	5	134	2	35
Clinical stage	0	28	–	15	–
	I	128	–	15	–
	II	97	–	15	–
	III	16	–	15	–
	IV	0	–	14	–
	NA	2	–	0	–
Mammogram category	1	12	105	5	25
	2	4	52	4	13
	3	41	9	10	3
	4	110	0	22	0
	5	94	0	24	0
	NA	10	132	9	34
Breast density	Fatty	18	1	2	1
	Scattered	127	68	36	18
	Heterogeneous dense	106	95	27	21
	Extremely dense	4	0	1	1
	NA	16	134	8	34
Histological grade	1	81	–	13	–
	2	118	–	27	–
	3	47	–	23	–
	NA	25	–	11	–

Data are presented as median (interquartile range) for continuous variables and number for categorical variables. Baseline characteristics are presented descriptively, and no significance tests were performed for between-group comparisons. Abbreviations: IQR, interquartile range; BC, breast cancer.

Based on the batch-corrected expression data of 114 miRNAs in the training set, the following 12 individual classification algorithms were trained: (1) Logistic Regression with L1 regularization (Lasso), (2) Logistic Regression with L2 regularization (Ridge), (3) Logistic Regression with ElasticNet regularization, (4) Linear Support Vector Machine (Linear SVM), (5) Support Vector Machine with radial basis function kernel (SVM), (6) Nu-Support Vector Machine (Nu-SVM), (7) Nystroem kernel approximation with Support Vector Machine (Nystroem SVM), (8) Gradient Boosting Classifier, (9) Extreme Gradient Boosting (XGBoost), (10) Light Gradient Boosting Machine (LightGBM), (11) Histogram-based Gradient Boosting Machine (HGBM), and (12) Adaptive Boosting (AdaBoost).

To assess generalization performance while minimizing overfitting bias and to identify the optimal ensemble configuration, a 5-fold nested cross-validation (nested-CV) framework was applied within the training set. The five outer folds were generated by stratified splitting to maintain the proportion of breast cancer cases and non-cancer controls, using a fixed random seed for reproducibility. Within each outer training partition, an additional 5-fold stratified inner CV was used for hyperparameter optimization. Algorithms with relatively small search spaces were tuned by exhaustive grid search, whereas algorithms with larger search spaces were tuned using Optuna with the Tree-structured Parzen Estimator. Candidate hyperparameter configurations were evaluated using mean log loss across the inner folds, and the configuration with the lowest mean log loss was selected. StandardScaler was fitted using only the training data within each CV stage and then applied to the corresponding validation data.

To identify the optimal ensemble, all combinations of the 12 algorithms were systematically evaluated. The algorithms were grouped into three categories, Linear Models, Kernel-based SVM Classifiers, and Tree-based Gradient Boosting Methods, and only combinations including at least one algorithm from each group were considered to ensure model diversity. For each eligible combination, the predicted probabilities of breast cancer from each constituent algorithm were averaged to produce an ensemble output, hereafter referred to as the prediction score, as a continuous value ranging from 0 to 1, reflecting the likelihood of malignancy. The nested-CV AUC used for ensemble selection was calculated as the mean of the AUCs across the five outer folds. The out-of-fold predictions from the five outer folds were combined and used to calculate the pooled training-set AUC, sensitivity, specificity, accuracy, stage-specific sensitivity, and prediction-score distribution shown in Additional file: Fig. 2b. The combination with the highest mean outer-fold AUC was selected as the final model, comprising five algorithms: Lasso, Ridge, SVM, Nu-SVM, and HGBM. Final hyperparameters were selected by repeating the same inner-CV search procedure using the entire training set, rather than by averaging or voting across the outer-fold-specific values. Each constituent algorithm was then retrained on the entire training set, and the prediction score was computed by averaging the predicted probabilities of the five retrained algorithms.

Model performance was then assessed on the held-out test set, which was not used for model fitting or hyperparameter selection. The test set was intentionally enriched for each clinical stage to enable stage-stratified evaluation of model performance. As described above, however, ComBat batch correction had been performed jointly using the training and test samples.

Additional details regarding the hyperparameter search spaces, final selected settings, random seed handling, optimization trial numbers, and preprocessing within cross-validation are provided in the Additional Methods and Additional file: Table 6. All model development and evaluation procedures were implemented in Python version 3.13 using scikit-learn version 1.8.0 [25]. Hyperparameter optimization for models with larger search spaces was performed using Optuna version 4.6.0 [26] with Bayesian optimization based on the Tree-structured Parzen Estimator algorithm.

### Clinical staging

Clinical stage was determined according to the Union for International Cancer Control (UICC) TNM Classification of Malignant Tumors, 8th edition [27]. Clinical stages were assigned by breast surgeons at each participating institution based on clinical and imaging findings at diagnosis, prior to definitive treatment.

### Mammography assessment and breast composition

Mammographic findings were assessed according to the Japanese mammographic diagnostic categories, as defined by national breast cancer screening guidelines, and classified into categories 1–5 [28]. Mammograms were interpreted by breast surgeons at each participating institution.

Breast composition (breast density) was evaluated by visual assessment in accordance with the classification issued by the Japan Central Organization on Quality Assurance of Breast Cancer Screening, and categorized as fatty, scattered fibroglandular density, heterogeneously dense, or extremely dense [29].

### Immunohistochemistry and pathological assessment

Immunohistochemical evaluation of estrogen receptor (ER), progesterone receptor (PgR), human epidermal growth factor receptor 2 (HER2), and Ki-67 status was performed at each participating institution and interpreted by pathologists at each site, without central review.

ER and PgR status were classified as negative when < 10% and positive when ≥ 10% of invasive tumor cell nuclei were stained. Although international guidelines define hormone receptor positivity at a threshold of ≥ 1% [30, 31], since tumors with low hormone receptor expression have been reported to exhibit clinicopathological characteristics closer to hormone receptor–negative disease [31], a 10% cutoff was prespecified in this study.

HER2 status was defined as negative in cases with IHC scores of 0 or 1+, positive for scores of 3+, and determined by in situ hybridization for 2 + cases, in accordance with established guidelines [32].

Histological grade was assessed using the Nottingham grading system (Elston–Ellis modification of the Scarff–Bloom–Richardson system) [16].

The Ki-67 labeling index was dichotomized using a cutoff value of 20%. While the optimal cutoff for Ki-67 remains controversial, thresholds of approximately 20% have been widely adopted in surrogate subtype classification and clinical studies [33, 34].

Intrinsic subtype classification was performed based on hormone receptor status, HER2 status, and Ki-67 labeling index, as assessed at each participating institution. Tumors were classified as luminal A-like (ER-positive, HER2-negative, and Ki-67 ≤ 20%), luminal B-like (ER-positive, HER2-negative, and Ki-67 > 20%), luminal-HER2 (ER-positive and HER2-positive), HER2-enriched (ER-negative and HER2-positive), or triple-negative (ER-negative and HER2-negative), according to previously established clinical criteria.

### Statistical analysis

Statistical analyses other than model development were performed using R. Baseline characteristics of breast cancer cases and non-cancer controls were summarized descriptively using medians with interquartile ranges for continuous variables and counts for categorical variables. In accordance with reporting recommendations for descriptive baseline tables, significance tests were not used to compare baseline characteristics between groups.

To evaluate model discrimination, receiver operating characteristic (ROC) curves were generated, and area under the curve (AUC) values with 95% confidence intervals (CIs) were calculated using the DeLong method with the R “pROC” package. The 95% CIs for sensitivity and specificity were calculated using the bootstrap method with the same package.

Threshold-dependent performance was evaluated using a cross-validated cutoff derived within the training set using the Youden index and using fixed-specificity operating points. For the cross-validated cutoff, the Youden index was maximized separately in each held-out partition of the five outer folds, and the median of the five fold-specific cutoff values was adopted as the final cutoff. This cutoff was applied unchanged to the held-out test set. For the fixed-specificity analyses, thresholds corresponding to target specificities of 90% and 85% were determined from the pooled ROC curve based on the combined out-of-fold predictions in the training set and were then applied unchanged to the held-out test set. These fixed-specificity operating points were presented as descriptive performance summaries and were not used for model selection.

Exploratory subgroup analyses were performed to describe the distribution of model-derived prediction scores across clinical and imaging-related variables. To evaluate ordered trends in prediction scores across clinical stage and histological grade, the Jonckheere–Terpstra test was performed using the R “clinfun” package. Analyses according to age, menopausal status, TNM components, mammographic category, breast density, intrinsic tumor subtype, and collection center were interpreted descriptively as exploratory analyses.

All subgroup analyses were considered exploratory. Statistical testing was limited to the ordered trend analyses for clinical stage and histological grade, which addressed the prespecified secondary objective of evaluating associations between prediction scores and clinicopathological disease characteristics. Clinicopathological variables were not imputed. Participants with missing values for a given variable were excluded only from the corresponding subgroup analysis, whereas all available samples were retained for model development and overall performance evaluation.

## Results

### Patient characteristics and study cohort

Between November 2023 and October 2025, serum samples were collected from breast cancer patients and cancer-free controls across multiple participating institutions. After applying predefined inclusion and exclusion criteria, a total of 345 breast cancer cases from 10 centers and 373 control samples from three centers were included in the final analysis (Fig. 1). The clinicopathological characteristics of the study population are summarized descriptively in Table 1 and Additional file: Table 2. In the training set, breast cancer cases tended to be older than non-cancer controls. Among participants with available menopausal status data, breast cancer cases were more frequently postmenopausal than controls. Differences in mammographic breast density distribution were also observed between breast cancer cases and non-cancer controls in the training set. These imbalances were less apparent in the held-out test set.

### Comprehensive miRNA profiling and quality assessment

Comprehensive miRNA expression profiling of all serum samples was performed using the Ion GeneStudio S5 sequencing platform. Spike-in controls were used to monitor technical variability across the workflow from RNA extraction through sequencing, and the distribution of pairwise squared Pearson correlation coefficients (R²) calculated from spike-in expression profiles is shown in Additional file: Fig. 1a.

Principal component analysis performed on all 718 samples showed no pronounced visual clustering according to participating institution, sequencing batch, or dataset assignment (training vs. test set) (Additional file: Fig. 1b–d). No clear separation between breast cancer cases and cancer-free controls was observed in the first two principal components (Additional file: Fig. 1e). These quality-control analyses did not identify a dominant technical source of variation in the visualized principal component space.

### Overall diagnostic discrimination between breast cancer cases and controls

Of the miRNAs measured in serum samples, 114 miRNAs that were stably expressed in both breast cancer cases and cancer-free controls were selected as candidate features for model construction. Using the training set, an ensemble model was developed to leverage the complementary strengths of multiple base learners, thereby improving predictive accuracy and reducing the risk of overfitting to any single algorithm. The generalization performance of the model was evaluated through nested cross-validation within the training set (Table 2, Additional file: Fig. 2a–b). The mean AUC across the five outer validation folds was 0.826, whereas the pooled AUC calculated from the combined out-of-fold predictions was 0.823. The final model was subsequently evaluated in the held-out test set.

**Fig. 2:**
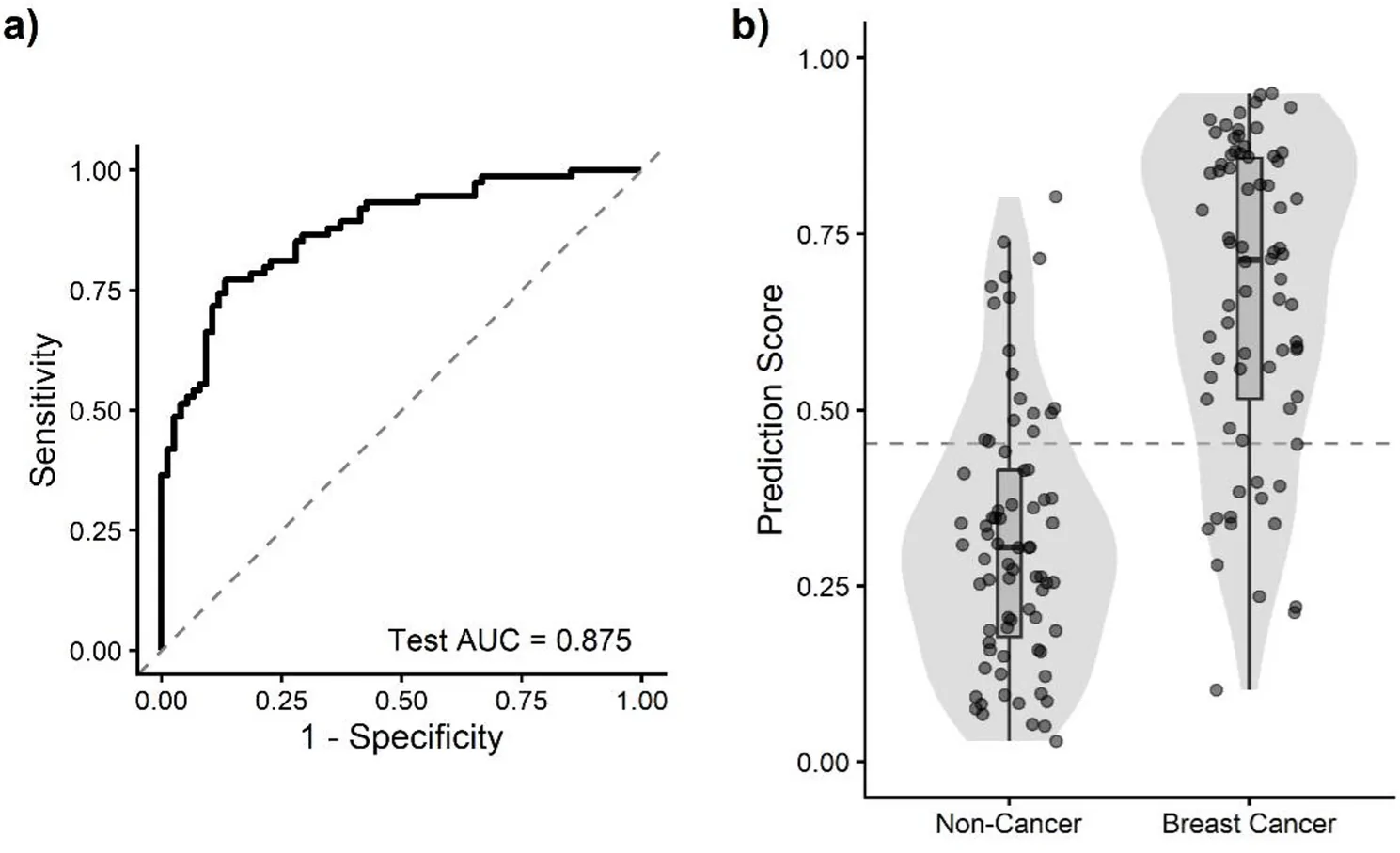
Diagnostic performance of the serum miRNA–based discrimination model. (**a**) Receiver operating characteristic (ROC) curve of the ensemble model for distinguishing breast cancer cases from non-cancer controls in the held-out test set. (**b**) Distribution of model-derived diagnostic prediction scores in breast cancer cases and non-cancer controls. Abbreviations: ROC, receiver operating characteristic; AUC, area under the curve

The ROC curve of the serum miRNA–based discrimination model is shown in Fig. 2a, demonstrating its ability to distinguish breast cancer cases from cancer-free controls, with an area under the curve (AUC) of 0.875 (95% confidence interval 0.820–0.930).

Figure 2b shows the distribution of model-derived diagnostic prediction scores in breast cancer cases and controls. Prediction scores were higher in breast cancer cases than in controls.

Diagnostic performance metrics based on the cross-validated cutoff are summarized in Table 2, and descriptive performance metrics at fixed-specificity operating points are presented in Additional file: Table 3. The final cutoff was derived within the training set by maximizing the Youden index separately in each held-out partition of the five outer folds and taking the median of the five fold-specific cutoff values. This cross-validated cutoff was then applied unchanged to the held-out test set. Using the resulting cutoff value of 0.453, the model achieved a sensitivity of 0.797 and a specificity of 0.773 in the held-out test set. The fixed-specificity thresholds were derived from the pooled ROC curve based on combined out-of-fold training predictions and were also applied unchanged to the held-out test set.

**Table 2:** Diagnostic performance of the serum miRNA–based discrimination model in the training and test sets

Performance metrics	Training set	Test set
AUC [95% CI]	0.823 [0.788**–**0.858]	0.875 [0.820**–**0.930]
Cross**–**validated threshold (Youden)	0.453 (median of five folds)	0.453 (same cutoff)
Sensitivity [95% CI]	0.768 [0.714**–**0.814]	0.797 [0.692**–**0.873]
Specificity [95% CI]	0.775 [0.724**–**0.819]	0.773 [0.667**–**0.853]
Accuracy [95% CI]	0.772 [0.735**–**0.804]	0.785 [0.713**–**0.844]

Note: The cross-validated cutoff was defined as the median of five fold-specific thresholds determined by maximizing the Youden index in the held-out partitions of the outer cross-validation folds. The resulting cutoff was applied unchanged to the held-out test set. AUC values are presented with 95% confidence intervals calculated using the DeLong method. Training-set AUC, sensitivity, specificity, and accuracy were calculated from the combined out-of-fold predictions generated across the five outer folds of nested cross-validation. Abbreviations: AUC, area under the curve; CI, confidence interval.

### Stage-specific subgroup analysis

**Fig. 3:**
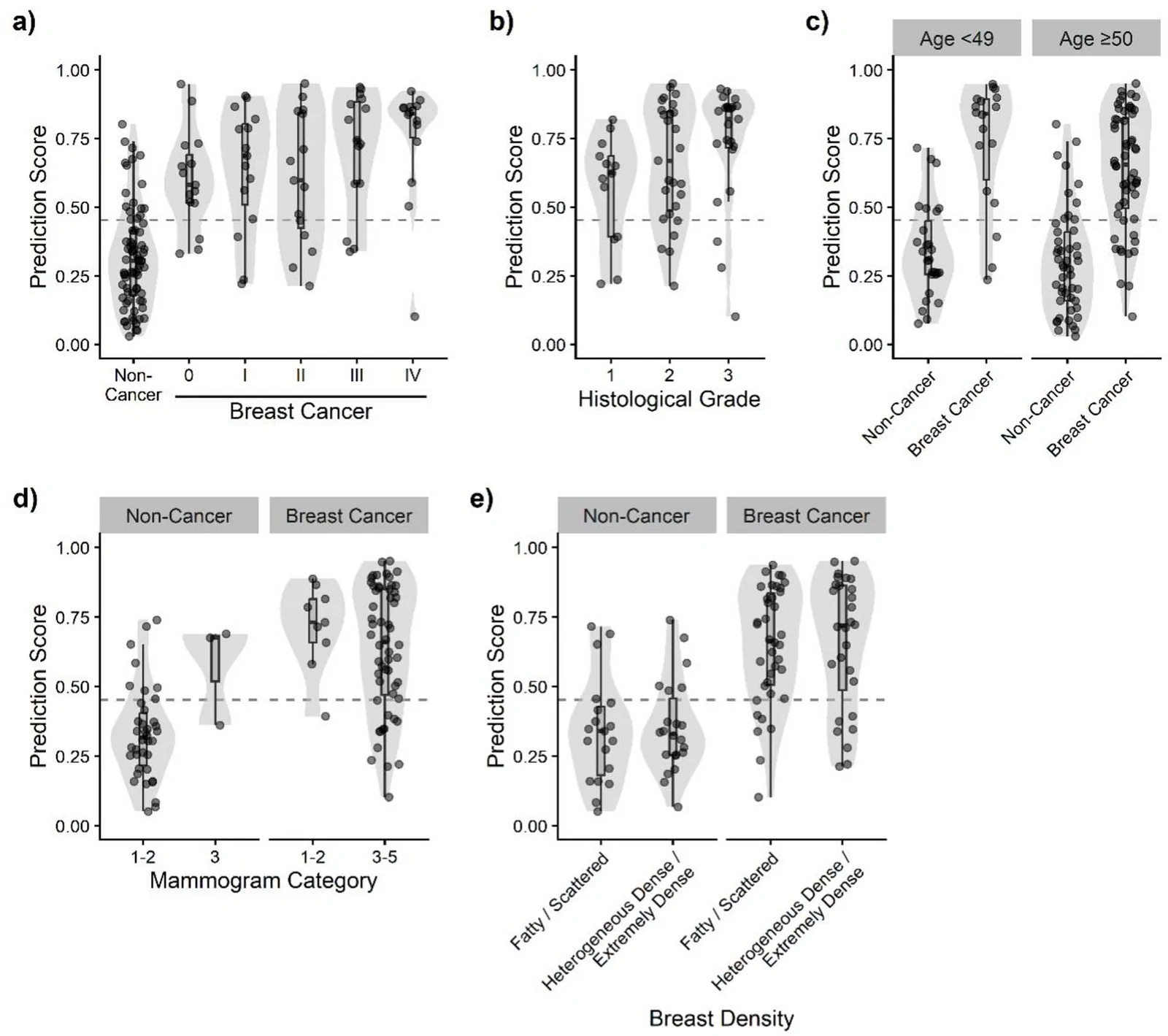
Distribution of model-derived prediction scores across clinical and imaging-related subgroups. (**a**) Clinical stage. Jonckheere–Terpstra test for an increasing trend: JT = 1338, *p* = 0.0102. (b) Histological grade. Jonckheere–Terpstra test for an increasing trend: JT = 822, *p* = 0.00836. (c) Age group (≤ 49 years vs. ≥ 50 years). (d) Mammographic diagnostic category (C1–2 vs. C3–5). (e) Breast density (low density: fatty or scattered fibroglandular density; high density: heterogeneously dense or extremely dense)

Stage-specific subgroup analyses were performed to evaluate the relationship between clinical stage and the model-derived diagnostic prediction score. As shown in Fig. 3a, prediction scores increased progressively with advancing clinical stage. An exploratory trend analysis using the Jonckheere–Terpstra test supported an ordered increase in prediction scores across clinical stages.

The analyses of clinical stage and histological grade were exploratory ordered-trend analyses. The remaining subgroup comparisons are presented descriptively.

Stage-specific diagnostic performance was further evaluated using sensitivity and specificity analyses. Detailed performance metrics for each clinical stage in the training and test sets are summarized in Table 3 and Additional file: Table 4. Due to the limited number of cases, patients with stage IV disease were not included in the training set, but were evaluated in the test set.

To further explore the contribution of individual staging components, additional analyses were conducted according to clinical T, N, and M categories (Additional file: Fig. 3a–c). Prediction scores tended to increase with higher cT categories and were higher in patients with distant metastasis, whereas no clear ordered trend was observed according to cN status.

**Table 3:** Stage-specific sensitivity of the serum miRNA–based discrimination model in the training and test sets

Stage	Training set sensitivity [95% CI]	Test set sensitivity [95% CI]
Stage 0	0.786 [0.605–0.898]	0.800 [0.548–0.930]
Stage I	0.734 [0.652–0.803]	0.800 [0.548–0.930]
Stage II	0.835 [0.749–0.896]	0.667 [0.417–0.848]
Stage III	0.563 [0.332–0.769]	0.800 [0.548–0.930]
Stage IV	NA	0.929 [0.685–0.987]

Note: Sensitivity and corresponding 95% confidence intervals were calculated for each stage using the cross-validated cutoff of 0.453, defined as the median of the five fold-specific thresholds derived within the training set. Training-set stage-specific sensitivities were calculated from the combined out-of-fold predictions, and the same cutoff was applied unchanged to the held-out test set. Abbreviations: CI, confidence interval; NA, not applicable.

### Additional subgroup analyses

Histological grade was also associated with model-derived diagnostic prediction scores. As shown in Fig. 3b, prediction scores showed an ordered increase across histological grades, supported by exploratory trend analysis using the Jonckheere–Terpstra test.

Age-stratified analyses were performed to explore whether model-derived prediction scores differed between breast cancer cases and cancer-free controls within age subgroups. When stratified by age (≤ 49 years vs. ≥ 50 years), prediction scores were higher in breast cancer cases than in controls in both age groups (Fig. 3c). Similar patterns were observed when analyses were stratified by menopausal status (Additional file: Fig. 4).

Prediction score distributions did not show a clear difference between the grouped mammographic categories of C1–2 and C3–5 (Fig. 3d). In the non-cancer group, most participants with available mammographic data were classified as category 1 or 2, whereas categories 4 and 5 were observed only among breast cancer cases. Despite these differences in category distribution between cases and controls, the model-derived prediction score did not show a clear relationship with the grouped mammographic categories.

Similarly, prediction score distributions were comparable between low-density breasts, defined as fatty or scattered fibroglandular density, and high-density breasts, defined as heterogeneously dense or extremely dense (Fig. 3e). Comparable patterns were observed in both breast cancer cases and non-cancer controls, suggesting that the model-derived prediction score was not strongly associated with mammographic breast density in this cohort.

Subgroup analyses were also performed to evaluate differences in model-derived diagnostic prediction scores across intrinsic tumor subtypes, based on tumor classification into luminal A-like, luminal B-like, luminal-HER2, HER2-enriched, and triple-negative subtypes, as described in the Methods section. Prediction score distributions were broadly comparable across intrinsic tumor subtypes (Additional file: Fig. 5), with no clear subtype-specific differences observed in this exploratory analysis. Subgroup analyses were also conducted to assess whether prediction scores varied across collection centers. It should be noted that the hospitals contributing non-cancer controls and those contributing breast cancer cases were different institutions. As shown in Additional file: Fig. 6, prediction score distributions did not show marked differences between centers within either the non-cancer group or the breast cancer group. These findings did not indicate substantial inter-institutional differences in model-derived prediction scores within each diagnostic group, although further external validation is required to confirm generalizability across collection sites.

## Discussion

In this multicenter study, we developed and validated a serum miRNA–based discrimination model for breast cancer using samples collected across multiple institutions. The model demonstrated favorable overall diagnostic performance in distinguishing breast cancer cases from cancer-free controls, as reflected by the AUC and corresponding sensitivity and specificity metrics. Importantly, diagnostic performance was evaluated independently from score behavior, allowing a structured interpretation of both discrimination accuracy and biological gradient.

A notable finding of the present study was the stage-associated increase in miRNA-derived diagnostic prediction scores. Prediction scores increased progressively with advancing clinical stage, and exploratory trend analysis supported an ordered increase across stages. This pattern suggests that circulating miRNA profiles captured by the model are associated with disease extent, rather than serving solely as a binary diagnostic indicator.

Histological grade was also associated with prediction score distribution. An ordered increase in prediction scores was observed across histological grades and was supported by exploratory trend analysis. This finding suggests that the model-derived score may be related to clinicopathological features associated with tumor aggressiveness. However, the observed relationship remains correlational and does not establish that the prediction score directly reflects biological aggressiveness.

Age-stratified analyses showed higher prediction scores in breast cancer cases than in controls in both younger and older groups, with similar patterns observed after stratification by menopausal status. Prediction scores did not show a clear systematic relationship with mammographic diagnostic category, despite marked differences in category distribution between cases and controls. Similarly, prediction score distributions were broadly comparable between low- and high-density breasts, suggesting that the circulating miRNA signal captured by the model was not strongly associated with imaging suspicion or mammographic breast density in this cohort. This finding may be clinically relevant because dense breast tissue can reduce mammographic sensitivity, whereas circulating molecular biomarkers are conceptually independent of radiographic tissue overlap. For contextual reference, the diagnostic performance reported for mammography and adjunctive ultrasonography in previous screening studies, together with that of the present serum miRNA–based model, is summarized in Additional file: Table 5 [7, 11, 35]; however, these estimates should not be interpreted as a direct head-to-head comparison because of differences in study design and population.

Several previous studies have investigated the diagnostic potential of circulating miRNAs in breast cancer. Previous reports have demonstrated that specific circulating miRNA panels can distinguish breast cancer cases from healthy controls with moderate to high accuracy [17–21]. These investigations provided important proof-of-concept evidence supporting the feasibility of blood-based miRNA diagnostics. However, many of these studies were conducted in relatively small or single-center cohorts, and primarily focused on binary case-control discrimination without detailed assessment of score behavior across clinicopathological strata.

Recent studies have demonstrated the expanding use of interpretable machine learning approaches in breast cancer research, including prediction of response to neoadjuvant chemotherapy and long-term clinical outcomes, as well as recurrence prediction after breast-conserving surgery for ductal carcinoma in situ. These studies integrated clinical, pathological, inflammatory, or imaging-derived features with machine learning models and used SHAP-based approaches to improve model interpretability [36, 37]. Unlike these imaging- or clinicopathology-based prediction studies, the present study focused on serum miRNA profiles as circulating molecular biomarkers for breast cancer detection and evaluated the association between model-derived prediction scores and clinicopathological disease characteristics. Our findings suggest that machine learning applied to blood-based molecular data may provide a complementary research direction to existing imaging- and clinicopathology-based prediction models, although further prospective validation is required.

In contrast, the present study incorporated a multicenter cohort and systematically examined the relationship between the miRNA-based prediction score and clinical stage. Prediction scores increased progressively with advancing clinical stage, and an exploratory trend analysis supported an ordered increase across stages. This stage-associated gradient suggests that the circulating miRNA signals captured by the model may be related not only to the presence of malignancy but also to disease extent. Such ordered score behavior has been less consistently examined in previous miRNA-based diagnostic studies and provides additional biological context for the observed discrimination performance. However, this association remains correlational and requires confirmation in independent cohorts.

Further exploratory analyses of individual TNM components provided additional context. Prediction scores tended to increase across higher cT categories and were generally higher among patients with distant metastasis, whereas no clear ordered pattern was observed across cN categories. These descriptive findings may suggest that circulating miRNA profiles are more closely associated with primary tumor burden and systemic disease status than with regional nodal involvement. However, given the exploratory nature of these analyses, the limited number of patients with stage IV disease, and the exclusion of stage IV cases from the training set, these findings should be interpreted cautiously and require further validation.

Despite its established role as the cornerstone of breast cancer screening, mammography has recognized diagnostic limitations. Population-based screening programs have reported variable sensitivity depending on screening conditions and patient populations, and mammographic performance is particularly influenced by breast density. In women with dense breast tissue, mammographic sensitivity is reduced, contributing to interval cancers and delayed cancer detection. This limitation is particularly relevant in East Asian populations, in whom dense breast tissue is common among screening-age women.

In the present study, the serum miRNA–based model demonstrated promising discrimination in a held-out test set. However, direct comparison between the present model and mammography should be interpreted cautiously because of differences in study design, target population, disease prevalence, and evaluation setting. In particular, the present case-control cohort does not directly represent a population-based screening cohort. Therefore, the findings should be interpreted as preliminary evidence supporting further investigation of serum miRNA profiling, rather than as evidence of established screening performance.

Importantly, the model-derived prediction score was not clearly influenced by mammographic category or breast density. This observation suggests that circulating miRNA profiles may provide biological information that differs from imaging-detectable structural features. Future prospective studies are needed to determine whether such information provides additive diagnostic value when combined with mammography or ultrasonography.

From a biological perspective, circulating miRNAs may originate from tumor cells, the tumor microenvironment, or host responses associated with malignancy. As disease extent increases, tumor-associated molecular signals in the circulation may also change, which may partly explain the observed stage-associated gradient in prediction scores. Nevertheless, the present study was not designed to determine the biological origin of individual miRNAs or to establish a causal relationship between tumor burden and prediction score.

The ordered score behavior observed in this study suggests that the serum miRNA–based prediction score may provide information beyond binary case-control discrimination. This characteristic differs conceptually from imaging-based detection, which relies primarily on visualization of structural abnormalities. However, the potential clinical role of circulating miRNA profiling should be evaluated in future prospective studies, particularly in relation to diagnostic thresholds, positive and negative predictive values, and additive value when combined with established imaging modalities.

Exploratory analyses across intrinsic tumor subtypes showed broadly comparable prediction score distributions among luminal A-like, luminal B-like, luminal-HER2, HER2-enriched, and triple-negative subtypes, with no clear subtype-specific pattern observed in this cohort. These observations suggest that the model-derived diagnostic signal was not clearly dominated by a particular intrinsic tumor subtype. However, given the exploratory nature of the analysis, the subgroup sizes, and the use of locally assessed pathological parameters, further evaluation in larger and more standardized cohorts is warranted.

Several limitations of this study merit consideration. First, this was an observational case-control study, and the breast cancer cases and cancer-free controls were not recruited from the same underlying screening population. Breast cancer cases were enrolled from multiple clinical institutions, whereas non-cancer controls were derived from previously collected control cohorts and were confirmed to have no cancer diagnosis within one year after blood collection. Therefore, the control group may have represented a relatively healthy population, and selection bias cannot be excluded. This design may have led to an optimistic estimate of diagnostic performance compared with that expected in an unselected real-world screening population.

Second, baseline characteristics differed between breast cancer cases and non-cancer controls in the training set, including age, menopausal status, and breast density. Although subgroup analyses by age, menopausal status, and breast density suggested that the model-derived prediction score was not solely driven by these factors, residual confounding cannot be excluded. Future prospective studies in representative screening cohorts, in which cases and controls are sampled from the same source population, will be required to clarify the generalizability and clinical applicability of the model.

Third, patients with a current or prior history of malignancy were excluded, and the specificity of the model relative to other cancer types or benign breast diseases was not evaluated. Therefore, the present results should not be interpreted as evidence of breast cancer specificity in broader clinical settings. Further validation in more heterogeneous populations, including individuals with other malignancies, benign breast conditions, and symptomatic patients undergoing diagnostic evaluation, will be necessary.

Fourth, ComBat batch correction was performed jointly across the training and test samples and included diagnosis as a covariate. Although the test set was not used for model fitting or hyperparameter selection, preprocessing was therefore not fully independent, and test-set diagnosis labels contributed to estimation of the batch-correction parameters. This information sharing may have led to an optimistic estimate of test-set performance. In addition, because collection center was not included in the ComBat model, residual site-related variation cannot be excluded if sequencing run and collection center were imbalanced. Future external validation should process the validation cohort independently using a prespecified harmonization strategy that does not use validation outcomes.

Other limitations include the retrospective analytical design, despite prospective sample collection according to predefined protocols. Pathological and immunohistochemical assessments, including Ki-67 evaluation and subtype classification, were performed locally at each participating institution without centralized review, which could have introduced inter-institutional variability. In addition, mammographic category classification and breast density assessment were performed according to Japanese clinical practice standards. While these criteria largely align with the BI-RADS framework, minor differences in categorization and interpretation may limit direct international comparability. Finally, although the model showed promising discrimination in the present held-out test set, its potential clinical role as an adjunct to imaging-based screening requires further prospective validation, including evaluation of positive and negative predictive values, clinical decision thresholds, and additive diagnostic value when combined with mammography or ultrasonography.

## Conclusion

In conclusion, this multicenter case-control study developed and validated a serum miRNA–based discrimination model that showed promising diagnostic performance for distinguishing breast cancer cases from cancer-free controls. The model-derived prediction score was associated with clinical stage and histological grade, suggesting that circulating miRNA profiles may capture biologically relevant information related to clinicopathological disease characteristics. However, because of the case-control design and differences between the study population and a real-world screening population, further prospective validation in representative screening cohorts is required. Rather than replacing imaging-based screening, serum miRNA profiling may warrant further investigation as a potential adjunctive diagnostic approach.

## Supplementary Information

Below is the link to the electronic supplementary material.

Supplementary Material 1

## Data Availability

The datasets generated and analyzed during the current study are not publicly available because they contain proprietary information related to the miRNA measurement platform, but may be available from the corresponding author on reasonable request, subject to institutional and contractual restrictions.

## Abbreviations

AUC: Area under the curve
CI: Confidence interval
cM: Clinical metastasis classification
cN: Clinical nodal classification
cT: Clinical tumor classification
ER: Estrogen receptor
HER2: Human epidermal growth factor receptor 2
HGBM: Histogram-based gradient boosting machine
IHC: Immunohistochemistry
miRNA: microRNA
NGS: Next-generation sequencing
Nu-SVM: Nu-support vector machine
PgR: Progesterone receptor
ROC: Receiver operating characteristic
RPM: Reads per million
SVM: Support vector machine
TNM: Tumor–node–metastasis classification
UICC: Union for International Cancer Control
