# Supplementary material for: Multicenter development and validation of a serum microRNA–based diagnostic model for breast cancer and its association with tumor burden and biological aggressiveness: an observational case-control study
Source: Breast Cancer Res. 2026 Aug 6;28:187. doi: 10.1186/s13058-026-02361-1 (PMC13729394; doi:10.1186/s13058-026-02361-1)
Supplement: Supplementary file 1 [file 13058_2026_2361_MOESM1_ESM.docx]

**Additional Material**

Multicenter Development and Validation of a Serum microRNA-Based Diagnostic Model for Breast Cancer and Its Association with Tumor Burden and Biological Aggressiveness: An Observational Case-Control Study

Ayane Yamaguchi et al.

**Additional Methods**

*Quality Control of RNA Extraction and miRNA Library Preparation*

QIAseq miRNA Library QC Spike-Ins (QIAGEN, 331535) were added to each sample prior to RNA extraction to monitor technical variability across the entire workflow, from RNA extraction through library preparation to sequencing. After sequencing, spike-in reads were mapped to reference sequences and normalized to RPM. Normalized values were subsequently log2-transformed. Within each sequencing run, Pearson correlation coefficients (r) were calculated for all sample pairs using the log2-transformed spike-in RPM profiles, and the coefficients were squared to obtain R² values. The distribution of the pairwise R² values across all sequencing runs was visualized as a histogram to evaluate inter-sample technical consistency.

*Pre-analytical sample handling*

Sample collection and serum preparation were performed according to a standardized internal protocol across participating institutions. Serum was aliquoted in 500-µL volumes and stored at −80°C. Samples were transported on dry ice on the day of shipment, with transport temperature monitored using data loggers. Samples underwent one freeze-thaw cycle at the time of RNA extraction.

*Principal Component Analysis*

Principal component analysis (PCA) was performed on the miRNA expression profiles of all samples (training and test sets combined, n = 718) to assess the overall structure of the data and potential sources of variation. Expression values for 114 miRNAs were used as input features. PCA was conducted using the prcomp function in R with centering and unit-variance scaling. The first two principal components (PC1 and PC2) were used for visualization. Samples were evaluated according to diagnosis (Non-cancer vs. Breast cancer), collection center, sequencing batch, and dataset (training set vs. test set) to determine potential batch effects and biological variations. A 95% confidence ellipse was overlaid for each group using a normal distribution assumption.

*Batch correction*

ComBat was applied to the log2(RPM + 1)-transformed expression data for 186 miRNAs across all 718 samples jointly. Sequencing run was specified as the batch variable, diagnosis (breast cancer vs. non-cancer) was included as a biological covariate, and collection center was not included in the correction model. Therefore, both training and test samples contributed to estimation of the batch-correction parameters.

*Model tuning and reproducibility*

The five outer folds were generated by stratified splitting to maintain the proportion of breast cancer cases and non-cancer controls, using random_state = 60. Within each outer training partition, hyperparameters were optimized using 5-fold stratified inner cross-validation. Grid search was used for algorithms with relatively small search spaces, whereas Optuna with the Tree-structured Parzen Estimator was used for algorithms with larger search spaces. Hyperparameter configurations were selected by minimizing mean log loss across the inner folds. Optuna was run for 200 trials in each outer fold and for 300 trials during final model construction. StandardScaler was fitted using only the training data within each cross-validation stage and applied to the corresponding validation data. Final selected hyperparameter values were obtained by repeating the same search procedure on the entire training set and were not calculated by averaging or voting across outer-fold-specific values. The detailed search spaces and final selected settings for the five constituent models are provided in Additional Table 6.

**Additional Figure Legends**

**Additional Figure 1**. Quality assessment and principal component analysis of circulating miRNA expression data.

(a) Distribution of pairwise squared Pearson correlation coefficients (R²) calculated from spike-in expression profiles across samples.
(b) Principal component analysis (PCA) colored by participating institution.
(c) PCA colored by sequencing batch and diagnosis.
(d) PCA colored by dataset assignment (training and test sets) and diagnosis.
(e) PCA colored by diagnosis.

No pronounced clustering was visually observed according to participating institution, sequencing batch, or dataset assignment after normalization and batch correction.

**Additional Figure 2.** Nested cross-validation performance of the serum miRNA–based diagnostic model

(a) Receiver operating characteristic curves for each outer validation fold of the 5-fold nested cross-validation, together with the pooled ROC curve based on the combined out-of-fold predictions.

(b) Distribution of pooled out-of-fold prediction scores in non-cancer controls and breast cancer cases.

Abbreviations: ROC, receiver operating characteristic; AUC, area under the curve.

**Additional Figure 3.** Distribution of model-derived probability scores according to TNM staging components

(a) Clinical T classification;

(b) Clinical N classification;

(c) Clinical M classification.

**Additional Figure 4.** Distribution of model-derived probability scores stratified by menopausal status

Probability score distributions in breast cancer cases and non-cancer controls stratified by menopausal status.

**Additional Figure 5.** Distribution of model-derived probability scores according to intrinsic tumor subtype

Tumors were classified into luminal A-like, luminal B-like, luminal-HER2, HER2-enriched, and triple-negative subtypes.

**Additional Figure 6**. Distribution of model-derived prediction scores across participating institutions.

Prediction scores are shown for non-cancer controls and breast cancer cases, stratified by participating institution (OCROMC, OPHACH, TOCROMC, KUHP, SGH, KTNH, and other institutions). No marked differences in prediction score distributions were observed across institutions within either diagnostic group. These findings are descriptive and do not establish generalizability across collection sites.

**Additional Figures**

**Additional Figure 1**

**Additional Figure 2**

**Additional Figure 3**

**Additional Figure 4**

**Additional Figure 5**

**Additional Figure 6**

**Additional Tables**

**Additional Table 1.** List of serum miRNAs used as candidate features for construction of the discrimination model

| miRNA | Accession | Sequence |
| --- | --- | --- |
| hsa-let-7a-5p/7c-5p | MIMAT0000062 /MIMAT0000064 | UGAGGUAGUAGGUUGUAUAGUU /UGAGGUAGUAGGUUGUAUGGUU |
| hsa-let-7b-5p | MIMAT0000063 | UGAGGUAGUAGGUUGUGUGGUU |
| hsa-let-7d-3p | MIMAT0004484 | CUAUACGACCUGCUGCCUUUCU |
| hsa-let-7d-5p | MIMAT0000065 | AGAGGUAGUAGGUUGCAUAGUU |
| hsa-let-7e-5p | MIMAT0000066 | UGAGGUAGGAGGUUGUAUAGUU |
| hsa-let-7f-5p | MIMAT0000067 | UGAGGUAGUAGAUUGUAUAGUU |
| hsa-let-7g-5p | MIMAT0000414 | UGAGGUAGUAGUUUGUACAGUU |
| hsa-let-7i-5p | MIMAT0000415 | UGAGGUAGUAGUUUGUGCUGUU |
| hsa-miR-1-3p | MIMAT0000416 | UGGAAUGUAAAGAAGUAUGUAU |
| hsa-miR-101-3p | MIMAT0000099 | UACAGUACUGUGAUAACUGAA |
| hsa-miR-103a-3p/107 | MIMAT0000101 /MIMAT0000104 | AGCAGCAUUGUACAGGGCUAUGA /AGCAGCAUUGUACAGGGCUAUCA |
| hsa-miR-106b-3p | MIMAT0004672 | CCGCACUGUGGGUACUUGCUGC |
| hsa-miR-10a-5p | MIMAT0000253 | UACCCUGUAGAUCCGAAUUUGUG |
| hsa-miR-10b-5p | MIMAT0000254 | UACCCUGUAGAACCGAAUUUGUG |
| hsa-miR-122-5p | MIMAT0000421 | UGGAGUGUGACAAUGGUGUUUG |
| hsa-miR-125a-5p | MIMAT0000443 | UCCCUGAGACCCUUUAACCUGUGA |
| hsa-miR-125b-5p | MIMAT0000423 | UCCCUGAGACCCUAACUUGUGA |
| hsa-miR-126-3p | MIMAT0000445 | UCGUACCGUGAGUAAUAAUGCG |
| hsa-miR-126-5p | MIMAT0000444 | CAUUAUUACUUUUGGUACGCG |
| hsa-miR-1277-5p | MIMAT0022724 | AAAUAUAUAUAUAUAUGUACGUAU |
| hsa-miR-128-3p | MIMAT0000424 | UCACAGUGAACCGGUCUCUUU |
| hsa-miR-1307-3p | MIMAT0005951 | ACUCGGCGUGGCGUCGGUCGUG |
| hsa-miR-130a-3p | MIMAT0000425 | CAGUGCAAUGUUAAAAGGGCAU |
| hsa-miR-140-3p | MIMAT0004597 | UACCACAGGGUAGAACCACGG |
| hsa-miR-140-5p | MIMAT0000431 | CAGUGGUUUUACCCUAUGGUAG |
| hsa-miR-141-3p | MIMAT0000432 | UAACACUGUCUGGUAAAGAUGG |
| hsa-miR-142-3p | MIMAT0000434 | UGUAGUGUUUCCUACUUUAUGGA |
| hsa-miR-142-5p | MIMAT0000433 | CAUAAAGUAGAAAGCACUACU |
| hsa-miR-143-3p | MIMAT0000435 | UGAGAUGAAGCACUGUAGCUC |
| hsa-miR-144-3p | MIMAT0000436 | UACAGUAUAGAUGAUGUACU |
| hsa-miR-144-5p | MIMAT0004600 | GGAUAUCAUCAUAUACUGUAAG |
| hsa-miR-145-5p | MIMAT0000437 | GUCCAGUUUUCCCAGGAAUCCCU |
| hsa-miR-146a-5p | MIMAT0000449 | UGAGAACUGAAUUCCAUGGGUU |
| hsa-miR-146b-5p | MIMAT0002809 | UGAGAACUGAAUUCCAUAGGCUG |
| hsa-miR-148a-3p | MIMAT0000243 | UCAGUGCACUACAGAACUUUGU |
| hsa-miR-148b-3p | MIMAT0000759 | UCAGUGCAUCACAGAACUUUGU |
| hsa-miR-150-5p | MIMAT0000451 | UCUCCCAACCCUUGUACCAGUG |
| hsa-miR-151a-3p | MIMAT0000757 | CUAGACUGAAGCUCCUUGAGG |
| hsa-miR-152-3p | MIMAT0000438 | UCAGUGCAUGACAGAACUUGG |
| hsa-miR-155-5p | MIMAT0000646 | UUAAUGCUAAUCGUGAUAGGGGUU |
| hsa-miR-15a-5p | MIMAT0000068 | UAGCAGCACAUAAUGGUUUGUG |
| hsa-miR-15b-5p | MIMAT0000417 | UAGCAGCACAUCAUGGUUUACA |
| hsa-miR-16-5p | MIMAT0000069 | UAGCAGCACGUAAAUAUUGGCG |
| hsa-miR-181a-5p | MIMAT0000256 | AACAUUCAACGCUGUCGGUGAGU |
| hsa-miR-182-5p | MIMAT0000259 | UUUGGCAAUGGUAGAACUCACACU |
| hsa-miR-183-5p | MIMAT0000261 | UAUGGCACUGGUAGAAUUCACU |
| hsa-miR-185-5p | MIMAT0000455 | UGGAGAGAAAGGCAGUUCCUGA |
| hsa-miR-186-5p | MIMAT0000456 | CAAAGAAUUCUCCUUUUGGGCU |
| hsa-miR-190a-5p | MIMAT0000458 | UGAUAUGUUUGAUAUAUUAGGU |
| hsa-miR-191-5p | MIMAT0000440 | CAACGGAAUCCCAAAAGCAGCUG |
| hsa-miR-192-5p/215-5p | MIMAT0000222 /MIMAT0000272 | CUGACCUAUGAAUUGACAGCC /AUGACCUAUGAAUUGACAGAC |
| hsa-miR-193a-5p | MIMAT0004614 | UGGGUCUUUGCGGGCGAGAUGA |
| hsa-miR-194-5p | MIMAT0000460 | UGUAACAGCAACUCCAUGUGGA |
| hsa-miR-195-5p | MIMAT0000461 | UAGCAGCACAGAAAUAUUGGC |
| hsa-miR-197-3p | MIMAT0000227 | UUCACCACCUUCUCCACCCAGC |
| hsa-miR-199a-3p/199b-3p | MIMAT0000232 /MIMAT0004563 | ACAGUAGUCUGCACAUUGGUUA /ACAGUAGUCUGCACAUUGGUUA |
| hsa-miR-199a-5p | MIMAT0000231 | CCCAGUGUUCAGACUACCUGUUC |
| hsa-miR-19a-3p | MIMAT0000073 | UGUGCAAAUCUAUGCAAAACUGA |
| hsa-miR-19b-3p | MIMAT0000074 | UGUGCAAAUCCAUGCAAAACUGA |
| hsa-miR-20a-5p | MIMAT0000075 | UAAAGUGCUUAUAGUGCAGGUAG |
| hsa-miR-20b-5p | MIMAT0001413 | CAAAGUGCUCAUAGUGCAGGUAG |
| hsa-miR-21-5p | MIMAT0000076 | UAGCUUAUCAGACUGAUGUUGA |
| hsa-miR-22-3p | MIMAT0000077 | AAGCUGCCAGUUGAAGAACUGU |
| hsa-miR-221-3p | MIMAT0000278 | AGCUACAUUGUCUGCUGGGUUUC |
| hsa-miR-223-3p | MIMAT0000280 | UGUCAGUUUGUCAAAUACCCCA |
| hsa-miR-223-5p | MIMAT0004570 | CGUGUAUUUGACAAGCUGAGUU |
| hsa-miR-23a-3p/23b-3p | MIMAT0000078 /MIMAT0000418 | AUCACAUUGCCAGGGAUUUCC /AUCACAUUGCCAGGGAUUACCAC |
| hsa-miR-24-3p | MIMAT0000080 | UGGCUCAGUUCAGCAGGAACAG |
| hsa-miR-25-3p | MIMAT0000081 | CAUUGCACUUGUCUCGGUCUGA |
| hsa-miR-26a-5p | MIMAT0000082 | UUCAAGUAAUCCAGGAUAGGCU |
| hsa-miR-26b-5p | MIMAT0000083 | UUCAAGUAAUUCAGGAUAGGU |
| hsa-miR-27a-3p/27b-3p | MIMAT0000084 /MIMAT0000419 | UUCACAGUGGCUAAGUUCCGC /UUCACAGUGGCUAAGUUCUGC |
| hsa-miR-28-3p | MIMAT0004502 | CACUAGAUUGUGAGCUCCUGGA |
| hsa-miR-29a-3p | MIMAT0000086 | UAGCACCAUCUGAAAUCGGUUA |
| hsa-miR-29b-3p | MIMAT0000100 | UAGCACCAUUUGAAAUCAGUGUU |
| hsa-miR-29c-3p | MIMAT0000681 | UAGCACCAUUUGAAAUCGGUUA |
| hsa-miR-301a-3p | MIMAT0000688 | CAGUGCAAUAGUAUUGUCAAAGC |
| hsa-miR-30a-5p | MIMAT0000087 | UGUAAACAUCCUCGACUGGAAG |
| hsa-miR-30b-5p/30c-5p | MIMAT0000420 /MIMAT0000244 | UGUAAACAUCCUACACUCAGCU /UGUAAACAUCCUACACUCUCAGC |
| hsa-miR-30d-5p | MIMAT0000245 | UGUAAACAUCCCCGACUGGAAG |
| hsa-miR-30e-3p | MIMAT0000693 | CUUUCAGUCGGAUGUUUACAGC |
| hsa-miR-30e-5p | MIMAT0000692 | UGUAAACAUCCUUGACUGGAAG |
| hsa-miR-32-5p | MIMAT0000090 | UAUUGCACAUUACUAAGUUGCA |
| hsa-miR-320a-3p/320b/320c/320d/320e | MIMAT0000510 /MIMAT0005792 /MIMAT0005793 /MIMAT0006764 /MIMAT0015072 | AAAAGCUGGGUUGAGAGGGCGA /AAAAGCUGGGUUGAGAGGGCAA /AAAAGCUGGGUUGAGAGGGU /AAAAGCUGGGUUGAGAGGA /AAAGCUGGGUUGAGAAGG |
| hsa-miR-328-3p | MIMAT0000752 | CUGGCCCUCUCUGCCCUUCCGU |
| hsa-miR-335-5p | MIMAT0000765 | UCAAGAGCAAUAACGAAAAAUGU |
| hsa-miR-338-3p | MIMAT0000763 | UCCAGCAUCAGUGAUUUUGUUG |
| hsa-miR-340-5p | MIMAT0004692 | UUAUAAAGCAAUGAGACUGAUU |
| hsa-miR-342-3p | MIMAT0000753 | UCUCACACAGAAAUCGCACCCGU |
| hsa-miR-361-3p | MIMAT0004682 | UCCCCCAGGUGUGAUUCUGAUUU |
| hsa-miR-361-5p | MIMAT0000703 | UUAUCAGAAUCUCCAGGGGUAC |
| hsa-miR-363-3p | MIMAT0000707 | AAUUGCACGGUAUCCAUCUGUA |
| hsa-miR-374a-5p | MIMAT0000727 | UUAUAAUACAACCUGAUAAGUG |
| hsa-miR-378a-3p/378c/378d/378e | MIMAT0000732 /MIMAT0016847 /MIMAT0018926 /MIMAT0018927 | ACUGGACUUGGAGUCAGAAGGC /ACUGGACUUGGAGUCAGAAGAGUGG /ACUGGACUUGGAGUCAGAAA /ACUGGACUUGGAGUCAGGA |
| hsa-miR-423-5p | MIMAT0004748 | UGAGGGGCAGAGAGCGAGACUUU |
| hsa-miR-424-5p | MIMAT0001341 | CAGCAGCAAUUCAUGUUUUGAA |
| hsa-miR-425-3p | MIMAT0001343 | AUCGGGAAUGUCGUGUCCGCCC |
| hsa-miR-425-5p | MIMAT0003393 | AAUGACACGAUCACUCCCGUUGA |
| hsa-miR-451a | MIMAT0001631 | AAACCGUUACCAUUACUGAGUU |
| hsa-miR-454-3p | MIMAT0003885 | UAGUGCAAUAUUGCUUAUAGGGU |
| hsa-miR-484 | MIMAT0002174 | UCAGGCUCAGUCCCCUCCCGAU |
| hsa-miR-486-5p | MIMAT0002177 | UCCUGUACUGAGCUGCCCCGAG |
| hsa-miR-532-5p | MIMAT0002888 | CAUGCCUUGAGUGUAGGACCGU |
| hsa-miR-584-5p | MIMAT0003249 | UUAUGGUUUGCCUGGGACUGAG |
| hsa-miR-590-3p | MIMAT0004801 | UAAUUUUAUGUAUAAGCUAGU |
| hsa-miR-625-3p | MIMAT0004808 | GACUAUAGAACUUUCCCCCUCA |
| hsa-miR-629-5p | MIMAT0004810 | UGGGUUUACGUUGGGAGAACU |
| hsa-miR-660-5p | MIMAT0003338 | UACCCAUUGCAUAUCGGAGUUG |
| hsa-miR-7-5p | MIMAT0000252 | UGGAAGACUAGUGAUUUUGUUGUU |
| hsa-miR-744-5p | MIMAT0004945 | UGCGGGGCUAGGGCUAACAGCA |
| hsa-miR-92a-3p | MIMAT0000092 | UAUUGCACUUGUCCCGGCCUGU |
| hsa-miR-93-5p | MIMAT0000093 | CAAAGUGCUGUUCGUGCAGGUAG |
| hsa-miR-98-5p | MIMAT0000096 | UGAGGUAGUAAGUUGUAUUGUU |
| hsa-miR-99b-5p | MIMAT0000689 | CACCCGUAGAACCGACCUUGCG |

**Additional Table 2.** Detailed clinicopathological characteristics of the study population

| Variables | | Training BC | Test BC |
| --- | --- | --- | --- |
| Sample size [n] | | 271 | 74 |
| cT | Tis | 27 | 13 |
|  | T0 | 1 | 2 |
|  | T1 | 134 | 16 |
|  | T2 | 94 | 18 |
|  | T3 | 8 | 14 |
|  | T4 | 5 | 11 |
|  | NA | 2 | 0 |
| cN | N0 | 230 | 42 |
|  | N1 | 26 | 14 |
|  | N2 | 5 | 4 |
|  | N3 | 8 | 14 |
|  | NA | 2 | 0 |
| cM | M0 | 269 | 60 |
|  | M1 | 0 | 14 |
|  | NA | 2 | 0 |
| Estrogen receptor | Negative | 43 | 16 |
|  | Positive | 212 | 51 |
|  | NA | 16 | 7 |
| Progesterone receptor | Negative | 78 | 25 |
|  | Positive | 172 | 40 |
|  | NA | 21 | 9 |
| HER2 | Negative | 208 | 50 |
|  | Positive | 33 | 12 |
|  | NA | 23 | 11 |
| Ki–67 | Low | 126 | 23 |
|  | High | 120 | 41 |
|  | NA | 25 | 10 |
| Subtype | Luminal A–like | 111 | 21 |
|  | Luminal B–like | 66 | 20 |
|  | HR+/HER2+ | 22 | 7 |
|  | HR–/HER2+ | 10 | 5 |
|  | Triple Negative | 28 | 9 |
|  | NA | 34 | 12 |

**Additional Table 3.** Diagnostic performance at fixed-specificity operating points derived from pooled out-of-fold training predictions

| Performance metrics | Training set | | Test set | |
| --- | --- | --- | --- | --- |
| AUC [95% CI] | 0.823 [0.788–0.858] | | 0.875 [0.820–0.930] | |
| Threshold method | Sp90% | Sp85% | Sp90% | Sp85% |
| Threshold value | 0.610 | 0.558 | 0.610 | 0.558 |
| Sensitivity [95% CI] | 0.598  [0.538–0.654] | 0.653  [0.595–0.707] | 0.595  [0.481–0.699] | 0.716  [0.605–0.806] |
| Specificity [95% CI] | 0.899  [0.860–0.929] | 0.849  [0.804–0.885] | 0.907  [0.820–0.954] | 0.893  [0.803–0.945] |
| Accuracy [95% CI] | 0.756  [0.719–0.789] | 0.756  [0.719–0.789] | 0.752  [0.677–0.814] | 0.805  [0.734–0.861] |

Fixed-specificity thresholds corresponding to target specificities of 90% and 85% were derived from the pooled ROC curve based on combined out-of-fold predictions in the training set and applied unchanged to the test set. Training-set metrics were calculated from the same combined out-of-fold predictions.

**Additional Table 4.** Stage-specific sensitivity at fixed-specificity operating points derived from pooled out-of-fold training predictions

| Threshold method | Stage | Training set  sensitivity [95% CI] | Test set  sensitivity [95% CI] |
| --- | --- | --- | --- |
|  |  |  |  |
| Sp90% | Stage 0 | 0.643 [0.458–0.793] | 0.467 [0.248–0.699] |
|  | Stage I | 0.555 [0.468–0.638] | 0.600 [0.357–0.802] |
|  | Stage II | 0.649 [0.550–0.737] | 0.467 [0.248–0.699] |
|  | Stage III | 0.500 [0.280–0.720] | 0.667 [0.417–0.848] |
|  | Stage IV | NA | 0.786 [0.524–0.924] |
| Sp85% | Stage 0 | 0.679 [0.493–0.832] | 0.600 [0.357–0.802] |
|  | Stage I | 0.602 [0.515–0.682] | 0.733 [0.480–0.891] |
|  | Stage II | 0.732 [0.636–0.810] | 0.600 [0.357–0.802] |
|  | Stage III | 0.500 [0.280–0.720] | 0.800 [0.548–0.930] |
|  | Stage IV | NA | 0.857 [0.601–0.960] |

Stage-specific sensitivities were calculated using the same training-derived fixed-specificity thresholds. Training-set estimates were based on combined out-of-fold predictions, and the thresholds were applied unchanged to the test set.

**Additional Table 5.** Contextual summary of reported diagnostic performance of imaging–based screening modalities and the present serum miRNA-based model

Note: The performance estimates were derived from studies with different designs, populations, disease prevalences, and diagnostic settings. They are presented to provide clinical context and should not be interpreted as a direct head-to-head comparison.

| Modality | Population | Sensitivity | Specificity | Reference |
| --- | --- | --- | --- | --- |
| Mammography screening | Population screening | 75–90% | ~90–95% | Nelson 2016 |
| Mammography (dense breast) | Dense breast | ~50–65% | – | Mann 2022 |
| Mammography + ultrasound | Dense breast screening | 77% | 91% | Ohuchi 2016 |
| Serum miRNA model | Present study | 80% | 77% | Present study |

**Additional Table 6**
Hyperparameter search spaces and final selected settings for the five constituent models

| **Model** | **Hyperparameter** | **Search method** | **Search range** | **Final selected value** |
| --- | --- | --- | --- | --- |
| Common (all models) | Preprocessing | Fixed | StandardScaler | StandardScaler |
| Common (all models) | Optimization metric | Fixed | Log loss | Log loss |
| Common (all models) | Inner CV | Fixed | 5-fold stratified CV; shuffle=True; random_state=60 | 5-fold stratified CV; shuffle=True; random_state=60 |
| Common (all models) | Outer CV | Fixed | Five predefined stratified folds | Five predefined stratified folds |
| Common (all models) | Random seed | Fixed | random_state=60 | random_state=60 |
| Logistic regression (L1) | Penalty | Fixed | L1 | L1 |
| Logistic regression (L1) | C | Grid search | 0.001, 0.005, 0.01, 0.05, 0.1, 0.5, 1.0, 5.0, 10.0, 50.0, 100.0 | 0.5 |
| Logistic regression (L1) | Solver | Grid search | liblinear, saga | saga |
| Logistic regression (L1) | max_iter | Grid search | 1000, 3000, 5000 | 1000 |
| Logistic regression (L1) | tol | Grid search | 1e-5, 1e-4, 1e-3 | 0.001 |
| Logistic regression (L1) | class_weight | Grid search | None, balanced, {0:1, 1:2}, {0:1, 1:3} | None |
| Logistic regression (L1) | fit_intercept | Grid search | True, False | True |
| Logistic regression (L2) | Penalty | Fixed | L2 | L2 |
| Logistic regression (L2) | C | Grid search | 0.001, 0.005, 0.01, 0.05, 0.1, 0.5, 1.0, 5.0, 10.0, 50.0, 100.0, 500.0, 1000.0 | 0.1 |
| Logistic regression (L2) | Solver | Grid search | liblinear, lbfgs, saga | lbfgs |
| Logistic regression (L2) | max_iter | Fixed | 1000 | 1000 |
| Logistic regression (L2) | tol | Grid search | 1e-4, 1e-5 | 1e-5 |
| Logistic regression (L2) | class_weight | Grid search | None, balanced | None |
| Logistic regression (L2) | fit_intercept | Grid search | True, False | True |
| SVC + CalibratedClassifierCV | C | Optuna (TPE) | 1e-3 to 1e3 (log-uniform) | 0.001846 |
| SVC + CalibratedClassifierCV | kernel | Optuna (TPE) | rbf, poly, sigmoid | poly |
| SVC + CalibratedClassifierCV | tol | Optuna (TPE) | 1e-5 to 1e-3 (log-uniform) | 5.55e-5 |
| SVC + CalibratedClassifierCV | gamma | Optuna (TPE) | scale, auto (rbf/poly/sigmoid) | scale |
| SVC + CalibratedClassifierCV | degree | Optuna (TPE) | 2 to 5 (poly kernel only) | 4 |
| SVC + CalibratedClassifierCV | coef0 | Optuna (TPE) | 0.0 to 10.0 (poly/sigmoid kernel only) | 8.244 |
| SVC + CalibratedClassifierCV | probability | Fixed | True | True |
| SVC + CalibratedClassifierCV | Calibration method | Optuna (TPE) | sigmoid, isotonic | isotonic |
| SVC + CalibratedClassifierCV | Calibration CV folds | Optuna (TPE) | 3 to 5 | 4 |
| NuSVC + CalibratedClassifierCV | nu | Optuna (TPE) | 0.01 to 1.0 | 0.485 |
| NuSVC + CalibratedClassifierCV | kernel | Optuna (TPE) | rbf, poly, sigmoid | rbf |
| NuSVC + CalibratedClassifierCV | tol | Optuna (TPE) | 1e-5 to 1e-3 (log-uniform) | 2.88e-5 |
| NuSVC + CalibratedClassifierCV | gamma | Optuna (TPE) | scale, auto (rbf/poly/sigmoid) | scale |
| NuSVC + CalibratedClassifierCV | degree | Optuna (TPE) | 2 to 5 (poly kernel only) | 3 |
| NuSVC + CalibratedClassifierCV | coef0 | Optuna (TPE) | 0.0 to 10.0 (poly/sigmoid kernel only) | 0 |
| NuSVC + CalibratedClassifierCV | probability | Fixed | True | True |
| NuSVC + CalibratedClassifierCV | Calibration method | Optuna (TPE) | sigmoid, isotonic | sigmoid |
| NuSVC + CalibratedClassifierCV | Calibration CV folds | Optuna (TPE) | 3 to 5 | 5 |
| HistGradientBoostingClassifier | max_iter | Optuna (TPE) | 50 to 500 | 108 |
| HistGradientBoostingClassifier | learning_rate | Optuna (TPE) | 0.01 to 0.3 | 0.0406 |
| HistGradientBoostingClassifier | max_depth | Optuna (TPE) | 3 to 15 | 6 |
| HistGradientBoostingClassifier | max_leaf_nodes | Optuna (TPE) | 10 to 300 | 147 |
| HistGradientBoostingClassifier | min_samples_leaf | Optuna (TPE) | 5 to 100 | 22 |
| HistGradientBoostingClassifier | l2_regularization | Optuna (TPE) | 0 to 1.0 | 0.824 |
| HistGradientBoostingClassifier | max_bins | Optuna (TPE) | 32 to 255 | 100 |
| HistGradientBoostingClassifier | loss | Fixed | log_loss | log_loss |

Final selected values were obtained by repeating hyperparameter optimization on the entire training set using the same 5-fold inner-cross-validation scheme. Standardization parameters were estimated within each training partition.

TPE, Tree-structured Parzen Estimator.
